# Gender differences in psychosocial status of adolescents during COVID-19: a six-country cross-sectional survey in Asia Pacific

**DOI:** 10.1186/s12889-021-12098-5

**Published:** 2021-11-04

**Authors:** Jun Wang, Alec Aaron, Anurima Baidya, Christabel Chan, Erica Wetzler, Kevin Savage, Michael Joseph, Yunhee Kang

**Affiliations:** 1 Johns Hopkins School of Education, Baltimore, MD USA; 2 Johns Hopkins Bloomberg School of Public Health, Baltimore, MD USA; 3 World Vision Asia Pacific Regional Office, Singapore, Republic of Singapore; 4 World Vision United States, Federal Way, WA USA; 5 World Vision International, Geneva, Switzerland

**Keywords:** Adolescents, Psychosocial status, COVID-19, Asia-Pacific region, Gender

## Abstract

**Background:**

School closures and family economic instability caused by the COVID-19 lockdown measures have threatened the mental health and academic progress of adolescents. Through secondary data analysis of World Vision Asia Pacific Region’s COVID-19 response-assessments in May–June 2020, this study examined whether adolescents’ study, physical, and leisure activities, psychosocial status, and sources of COVID-19 information differed by gender.

**Methods:**

The assessments used cross-sectional surveys of adolescents in poor communities served by World Vision (*n* = 5552 males and *n* = 6680 females) aged 10–18 years old in six countries. The study households of adolescents were selected either by random sampling or non-probability convenience sampling and assessed using telephone or in-person interviews. Multivariate logistic regression analyses examined the relationship between gender and psychosocial status; daily activities (e.g., play, study); and sources of information about COVID-19.

**Results:**

Participation in remote education was low (range: 0.5–20.7% across countries), with gender difference found only in Vietnam. Compared to males, female adolescents were less likely to play physically with a range of AOR: 0.36–0.55 (*n* = 5 countries) or play video games with a range of AOR: 0.55–0.72 (*n* = 2 countries). Female adolescents were more likely to feel isolated or stressed (India, AOR = 1.13, 95%CI:1.00, 1.26); feel unsafe (the Philippines, AOR = 2.22, 95%CI:1.14, 4.33; Vietnam, AOR = 1.31, 95%CI:1.03, 1.47); be concerned about education (India, AOR = 1.24, 95%CI:1.09, 1.41; Myanmar, AOR = 1.59, 95%CI:1.05, 2.40); or be concerned about household income (India, AOR = 1.13, 95%CI:1.00, 1.28; Vietnam, AOR = 1.31, 95%CI:1.09, 1.58). Female adolescents were also less likely to obtain COVID-19 related information through internet/social media (Bangladesh, AOR = 0.51, 95%CI:0.41, 0.64; India, AOR = 0.84, 95%CI:0.73, 0.96; and Myanmar, AOR = 0.65, 95%CI:0.43, 0.97) and mobile call or short message (India, AOR = 0.88, 95%CI:0.80, 0.98) but more likely to get the information from friends (Vietnam, AOR = 1.18, 95%CI:1.02, 1.36) and family (Bangladesh, AOR = 1.44, 95% CI:1.21, 1.70; India, AOR = 1.29, 95% CI:1.15, 1.45).

**Conclusions:**

An understanding of gender differences in the impacts of COVID-19 on adolescents' schooling, physical, and mental health can inform adolescent protection interventions. Psychosocial support during response and recovery phases needs to pay special attention to gender differences, since female adolescents’ psychosocial status is at higher risk when facing the challenges of this pandemic.

**Supplementary Information:**

The online version contains supplementary material available at 10.1186/s12889-021-12098-5.

## Background

The COVID-19 pandemic has caused unprecedented changes to daily life, affecting the operation of everything, from economic markets to local education systems [1]. As of early September 2021, estimates project there have been over 200 million COVID-19 cases globally, with 4.5 million deaths [2]. The countries in the Asia Pacific region have been acutely impacted, representing a large proportion of the overall disease burden, with an estimated nearly 55 million cases and 850 thousand deaths [3].

Globally, the COVID-19 pandemic has negatively impacted the mental and physical health of caregivers, both familial and medical professionals. Healthcare workers, in particular, are exposed to novel job stressors that strongly deteriorate job satisfaction, life satisfaction, and heighten turnover rates [4, 5]. A recent systematic review of Asia Pacific countries found that over one-quarter of healthcare providers may have experienced depression, or anxiety, as a direct result of this pandemic [6]. Other studies from this region report these rates to be even greater, with increasing intensity among frontline workers [7–9]. The psychological and mental health consequences of COVID-19 are clear among healthcare professionals, but these tribulations are not unique to this employment category [10]. The general public has also witnessed a dramatic rise in the prevalence of mental illness [11–13].

In the United States, a survey from the Centers for Disease Control and Prevention found a 3 to 4 times increase in the rates of adverse mental or behavioral health conditions relative to 1 year prior among surveyed adults; and suicidal ideation was significantly higher among younger adults (18–24 years) group and unpaid adult-caregivers group [14]. Caregivers with children or adolescents who need special health care may also face increased stress [15].

As part of the restrictions on travel, business operations, social gatherings, or so-called “lockdowns” in response to the pandemic, most countries around the world have also temporarily shut down schools over the last year [16]. As schools closed their doors, education shifted from primarily being in-person to taking place within the home. Such a shift may change adolescents’ learning behavior and have impacts on their self-efficacy and academic performance [17]. This has impacted more than 1.6 billion adolescents, or approximately 80% of the world’s students [18].

Beyond education, the pandemic is having a substantial impact on adolescents’ mental health. This is particularly concerning as adolescence is a period of heightened sensitivity to stress [19]. A meta-analysis showed a higher prevalence of anxiety, depression, and stress during COVID-19 pandemic than those reported before pandemic among college students [20]. Stressors arising from the pandemic, include, in addition to worries about education disruption, the restrictions on movement and social gathering, infection fears, financial loss, inadequate supplies or information, and stigma, can lead to a variety of negative psychological effects ranging from anxiety and depression to suicidal deaths [21]. One bright side of social isolation, allowing adolescents to spend more time with families, may serve as a source of mental balance if adolescents could receive adequate support from family members [22]. However, on the other hand, increased stressors faced by families (e.g., incoming loss, food insecurity, and parenting challenges) during the lockdown also may heighten children’s risk of neglect, exploitation, abuse, and violence [23–25]. In general, young adolescents may not have enough coping skills to deal with the negative psychosocial consequences [26]. Without proper early intervention, the negative impacts will extend to their later life [26, 27].

In addition, school closure cut off an important channel to receive information and knowledge related to COVID-19 for school-aged adolescents in Asian Pacific countries. Evidence showed COVID-19 related knowledge, which has been proved to be significantly associated with risk perception and precautionary behaviors [28], is important for adolescents to protect them from being infected by the pandemic. However, even in the US, where published guidelines were easily to be obtained by individuals, a substantial proportion of college students were found not washing their hands as recommended by the CDC [29]. It is plausible that adolescents in Asian Pacific countries may have insufficient information to practice protective behaviors.

The disruption of education has also exacerbated gender-based inequality [30]. School closures are leading to even higher gender disparity in dropout rates as female students face barriers related to sexual and reproductive health and gendered socioeconomic factors [31]. Gender inequality in education has long been an issue in some Asian countries, where boys are prioritized for educational opportunities over girls [32, 33] and past evidence from India also suggests that school-aged females are likely to have higher dropout rates [34]. As families face difficulties girls may be forced to take on additional household responsibilities to cope with the economic stress of the family [35]. This is primarily influenced through cultural and religion context according to a cross-section study [36].

Beginning in adolescence, females show greater negative responses to stress than males, which may be associated with differences in physiological response systems [37]. Other evidence suggests that women are more likely to cope stress with emotional and avoidance styles and suffer more from physical and psychological distress symptoms than males [38].

This study aims to examine if adolescents’ studying (i.e., studying at home, receiving remote education, and receiving online courses) and leisure activities (i.e., playing physically, watching TV, playing video games, and sleeping in daytime), psychosocial status, and sources of COVID-19 information differed by gender in Bangladesh, India, Indonesia, Myanmar, the Philippines, and Vietnam in the Asia Pacific Region. This paper describes the early experiences of adolescents with the COVID-19 pandemic. A better understanding of gender differences in the impacts of COVID-19 on adolescent’s schooling, physical, and mental health can inform policies and guide the improvement of adolescent protection interventions.

## Methods

### Data sources

This study is a secondary analysis of data drawn from a set of surveys of adolescent children conducted by World Vision (WV) as part of a multi-country “Rapid Recovery Assessment”. The assessment was carried out in 402 of the communities in which WV works across 13 Asia Pacific countries: Bangladesh, Cambodia, India, Indonesia, Laos, Mongolia, Myanmar, Nepal, Philippines, Sri Lanka, Thailand, Timor-Leste and Vietnam in May 2020. The assessment focused on the socio-economic impact of COVID-19 on vulnerable households and their children in order to guide WV’s continued programming to best support recovery from the effects of the pandemic.

The study uses data sets from the adolescent surveys in six of the 13 countries, which contains data of adolescents aged 10–18, who largely attended schools, from both urban and rural area, and excluding any respondents from 0 to 9 years of age. These surveyed a total of 12,232 adolescents (*n* = 5552 males; *n* = 6680 females): 1599 from Bangladesh, 5595 from India, 812 from Indonesia, 386 from Myanmar, 421 from the Philippines, and 3419 from Vietnam (Table 1). The other countries were excluded for various reasons. The Nepal survey collected significantly different information than other countries. The sample sizes in Cambodia (*n* = 238), Laos (*n* = 72), and Mongolia (*n* = 47) were too small. The Thailand survey was only administered to rural households. However, the present study considered the location of residence as a major confounding variable. The surveys in Sri-Lanka and Timor-Leste did not include adolescents.

**Table 1:** Demographic characteristics in Asia Pacific countries during early COVID-19

	Bangladesh ( *N *= 1599)	India ( *N* = 5595)	Indonesia ( *N* = 812)	Myanmar (*N* = 386)	Philippines (*N* = 421)	Vietnam (*N* = 3419)
	n (%)	n (%)	n (%)	n (%)	n (%)	n (%)
**Type of Community**						
Rural	1247 (77.99)	3871 (69.19)	733 (90.27)	80 (20.73)	311 (73.87)	3013 (88.13)
Urban	352 (22.01)	1724 (30.81)	79 (9.73)	306 (79.27)	110 (26.13)	406 (11.87)
**Gender**						
Male	701 (43.84)	2565 (45.84)	292 (35.96)	164 (42.49)	179 (42.52)	1651 (48.29)
Female	898 (56.16)	3030 (54.16)	520 (64.04)	222 (57.51)	242 (57.48)	1768 (51.71)
**Age**						
Younger Age (10–14 y)	485 (30.33)	2993 (53.49)	476 (58.62)	230 (59.59)	216 (51.31)	1986 (58.09)
Older Age (15–18 y)	1114 (69.67)	2602 (46.51)	336 (41.38)	156 (40.41)	205 (48.69)	1433 (41.91)
**Age, Mean (SD)**	15.33 (1.69)	14.31 (2.15)	13.70 (2.18)	14.09 (1.76)	14.43 (1.73)	14.27 (1.50)

Rural category included “Rural”, “Other” and “Tribal” communities. Urban category included “Urban”, “Semi-Urban” and “Slum” communities

### COVID-19 preventive measures in Asia Pacific region

In the Asia Pacific region, lockdown measures varied considerably between countries. In the first half of 2020, Bangladesh, the Philippines, and Vietnam had strict measures throughout the country; while in Indonesia and Myanmar, the measures were less strict and varied by locations [39–41]. In Myanmar, curfews were adopted by several regions, and a supplementary stay-at-home order was imposed by seven townships [40]. The Philippines experienced a long period of stay-at-home orders from mid-March to May and then again in August [42]. According to United Nations Educational, Scientific and Cultural Organization (UNESCO) [43], schools were closed country-wide until September 2020 in Bangladesh, India, Indonesia, Myanmar, and the Philippines. As a result, school-aged adolescents in the Asia-Pacific region faced substantial challenges and difficulties in daily life and to pursing education, though these differed between countries. One of the biggest issues, was availability and accessibility of temporary or emergency remote learning in lieu of physical school attendance. Governments in the Asia Pacific region responded with different strategies to mitigate the disadvantages brought by school closures. In Indonesia, India and Bangladesh, public broadcasters, using radio and television, were utilized to broadcast educational context to compensate the K-12 education [44, 45]. In Vietnam, adolescents returned to school from the beginning of May 2020, after more than 3 months’ social distancing measures [46]. In Myanmar, school started to reopen from July, with free face masks and shields provided to teachers and students [47]; however, with the second wave of COVID-19 cases, students were sent home again at the end of August 2020 [48]. UNESCO [49] estimates that on average 22 weeks’ school attendance has been lost so far across Eastern and South-Eastern Asia countries.

### Sampling methods

In all countries, the sampling frame was the population of children living within the boundaries of the Area Programmes (APs) of the WV office, which is the basic organizational unit for WV’s programming. It is a geographic area that consists of a community or set of communities within which WV collaborates for its long-term partnering to support sustainable development. All adolescent data came from a cross-sectional survey of households, within which a sub-set of adolescents were surveyed. Each country used different sampling approach and sample size determination, but largely, the sample size was aimed to have representative values for multiple variables at all sampled ADPs in each country. With some variation by country, available children in the selected households were all eligible to the child survey.

In Bangladesh, 50 households with children under 18 years old were selected randomly from each of 53 APs across 52 Upazilas of 24 districts from four regions. About 30 school going children (12–18 years of age) per AP were selected randomly for child survey. Thus total 1590 children were planned in this survey. If more than one child in a household, only the one enrolled in the Child Sponsorship Programming by World Vision would be interviewed.

In India, about 50 households from each of 111 APs and 30 household per seven special project areas were selected using non-probability convenience sampling. For the child survey, only households with children aged 10–18 years were included. Only one child per household was interviewed. If a household had more than one child, the interviewer interviewed a random child based on availability and comfort of that child.

In Indonesia, 247 out of 594 APs were selected, and a total of 20 adolescents were targeted in each AP in a way to get 10 respondents for households with 6–11 years of age and 10 respondents for households with 12–18 years of age. Only one child per household was interviewed. If a household has more than one child, interviewer asked for permission and willingness to the caregiver and the child about whom to be interviewed.

In Myanmar, a total of 31 APs from 46 districts in 13 of 14 States and Regions were randomly selected. Ten households were purposively sampled in each AP, with households hosting most vulnerable children (12–17 years old), children under 5 years old, pregnant and lactating women, children living with disabilities and Vision Fund Myanmar clients. If more than one child in households, the interview was made for older child; and if there were boys and girls in households, a boy was selected for the interview, according to the previous survey result that girls are mostly found than boys in a household.

In Vietnam, about 95 households (or 114 households based on AP who have 5 to 6 supervision areas) were randomly selected from 35 APs. If the interviewed household does not have any child aged 12–18 years or the child is absent from the survey location during the assessment, the enumerator selected a child from the nearest household in the COVID-19 Response participant list. If one household have more than one child aged 12–18 years, the interviewers interviewed both two children as they may have different opinions even they live in the same household.

In the Philippines, a total of 28 APs in Luzon, Visayas and Mindanao covering 229 barangays (the smallest administrative division in the Philippines) were selected. Only one child per household was interviewed. If more than one child in a household, the interview was made for a random child aged 12–17 years old based on availability and comfort.

### Data collection

The surveys used structured questionnaires that collected information from adolescents about their demographic characteristics (gender, age, residence); study and leisure activities; psychosocial status (feelings of happiness, unhappiness, isolation, stress, and concerns; and reasons for these); parental discipline; sources of COVID-19 information; perceptions of COVID-19; and their plans for after the lockdown.

Questionnaires were created in standard electronic document format (Microsoft Word or Excel) and translated into local languages. There was a master questionnaire, but additionally contextualized questions were included in each country. These were then transformed into electronic data-collection format using Kobo Toolbox. Data were then collected by interview and entered directly into Android tablets mostly through telephone conversation, or in-person when feasible, with appropriate COVID-19 protocols regarding social distancing and using personal protective equipment.

In each country, World Vision AP staff or non-AP staff were engaged in conducting phone surveys. The data collectors underwent training and pilot tests prior to collecting the information to become acquainted with the survey tools, using smartphones for data collection, and to understand the key objectives and aims of the assessment.

The interviewers provided the details of purpose and contents of the survey to the parents of the adolescent through mobile phones or household visits in remote areas where the covid-19 cases are few, and received verbal consent from the parents or legal guardians of adolescents (under 18 years old) and assent from adolescents themselves. All subjects completed the surveys voluntarily and the information collected via the survey was anonymized to maintain confidentiality. All methods were carried out in accordance with relevant guidelines and regulations.

### Available variables

#### Independent variables

The independent variable is adolescent gender (male vs. female).

#### Dependent variables

Questions assessing the impact of the lockdown on the adolescent mental health included whether the adolescent was feeling isolated or stressed and understanding any specific concerns the adolescent might have due to the stay-at-home orders. This included 1) study and playing activity during restriction; 2) negative psychosocial status; and 3) information sources of COVID-19.

The study and playing activity during restriction included 1) studying (i.e., all types of study activities), 2) remote education by school, 3) online courses, 4) playing-physically, 5) sleeping during daytime, 6) watching TV, and 7) playing games on TV, phones, and tablets. Since most schools were closed across these six countries when the data were collected in May 2020, variables describing the main daily activities of adolescents were collected. Adolescents were asked about how many hours they spent on each activity throughout the day.

Assessment of psychosocial status for being required to stay at home during COVID-19 included the following areas: 1) feeling isolated/stressed, 2) worrying about getting sick, 3) concerns about not going to school, 4) concerns about missing friends, 5) concerns about household income or food security, and 6) feeling unsafe or insecure.

The types of information source about COVID-19 included 1) internet/social media, 2) mobile phone (phone call/text), 3) friends, 4) family, and 5) TV.

#### Confounding variables

The confounding variables included in the regression models are type of residence (rural or urban), and young age (10–14 y) versus older age (15–18 y).

### Statistical analysis

Exploratory data analysis was conducted to present proportions for categorical variables and mean (standard deviation) for continuous variables. Chi- squared analysis was used to test difference for categorical variables and Student t-test for continuous variables. Odds ratios (OR), 95% confidence intervals (CI), and *p*-values were calculated through univariate and multivariable logistic regression of 1) the studying and leisure activity during restriction; 2) psychosocial status; and 3) information source on COVID-19 by adolescent gender. *P*-values less than 0.05 or 95% CI of odds ratios (ORs) not including 1.00 were considered to be statistically significant. Multivariable logistic regression was adjusted for type of residence (rural vs. urban) and adolescent age, and clustering at project area unit in each country. Stata 16.0 (Stata Corporation, College Station, TX, USA) was used for data management and statistical analysis.

## Results

### Demographic characteristics

The majority of adolescents in this study lived in rural area (69.2–90.3%) (Table 1). The number of female participants were slightly more than the males across these six countries from 51.7% in Vietnam to 64.0% in Indonesia. Bangladesh population had a larger proportion (69.7%) of individuals in the older age group (15–18 years old) than in the younger group (10–14 years old), while other countries had a greater proportion of younger adolescents (51.3–59.6%).

### Daily activity during restriction caused by COVID-19

#### Study activities

**Fig. 1:**
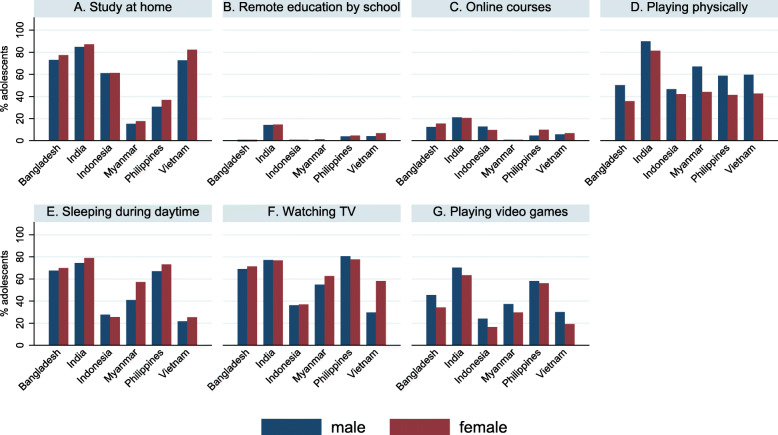
Daily activities done by adolescents in Asia Pacific countries during early COVID-19

Although the proportion of adolescents who studied in the past day varied by country (16.6–86.1%), the percentages of adolescents who received remote education (0.5–20.7%) or online courses (0.5–14.4%) were low across all six countries (Table 2 & Fig. 1). Adjusting for the type of community and age, in Vietnam, female adolescents had 1.73 times higher odds of spending time on study at home (AOR = 1.73, 95%CI:1.39, 2.16, *p* < 0.001), 1.23 times higher odds of having remote education (AOR = 1.23, 95%CI:1.03, 1.48, *p* = 0.02), and 1.74 times (AOR = 1.74, 95% CI:1.28, 2.37, p < 0.001) higher odds of having online courses during a day, compared to male adolescents. Female adolescents in Bangladesh and India also tended to spend more time on studying at home than their male peers, although the differences were not statistically significant (*p* = 0.08 & *p* = 0.05, respectively).

**Table 2:** Logistic regressions of adolescent gender for daily activities in Asia Pacific countries during early COVID-19

	Total	Male	Female	Male	Female	*p* value	Male	Female	*p* value
**Studying at home**	N (%)	n (%)	n (%)	COR	COR (95%CI)		AOR	AOR (95%CI)	
Bangladesh (*n* = 1599)	1206 (75.42)	512 (73.04)	694 (77.28)	1.00	1.26 (1.00, 1.57)	**0.046**	1.00	1.22 (0.98, 1.54)	0.08
India (*n* = 5595)	4815 (86.06)	2175 (84.80)	2640 (87.13)	1.00	1.21 (0.99, 1.49)	0.06	1.00	1.22 (0.998, 1.49)	0.05
Indonesia (*n* = 812)	496 (61.08)	178 (60.96)	318 (61.15)	1.00	1.01 (0.75, 1.35)	0.96	1.00	1.00 (0.75, 1.35)	0.98
Myanmar (*n* = 386)	64 (16.58)	25 (15.24)	39 (17.57)	1.00	1.18 (0.67, 2.10)	0.56	1.00	1.19 (0.68, 2.09)	0.54
Philippines (*n* = 421)	144 (34.20)	55 (30.73)	89 (36.78)	1.00	1.31 (0.82, 2.10)	0.26	1.00	1.37 (0.85, 2.21)	0.19
Vietnam (*n* = 3419)	2655 (77.65)	1201 (72.74)	1454 (82.24)	1.00	1.74 (1.38, 2.18)	**< 0.001**	1.00	**1.73 (1.39, 2.16)**	**< 0.001**
**Remote education by school**									
Bangladesh (*n* = 1599)	226 (14.13)	87 (12.41)	139 (15.48)	1.00	1.29 (0.87, 1.93)	0.21	1.00	1.24 (0.82, 1.86)	0.31
India(*n* = 5595)	1160 (20.73)	540 (21.05)	620 (20.46)	1.00	0.96 (0.84, 1.11)	0.61	1.00	0.96 (0.84, 1.11)	0.60
Indonesia (*n* = 812)	87 (10.71)	37 (12.67)	50 (9.62)	1.00	0.73 (0.46, 1.18)	0.20	1.00	0.76 (0.48, 1.21)	0.25
Myanmar (*n* = 386)	2 (0.52)	1 (0.61)	1 (0.45)	1.00	0.74 (0.05, 11.99)	0.83	1.00	0.76 (0.04, 13.29)	0.85
Philippines (*n* = 421)	32 (7.60)	8 (4.47)	24 (9.92)	1.00	2.35 (1.02, 5.41)	**0.044**	1.00	2.30 (0.96, 5.50)	0.06
Vietnam (*n* = 3419)	214 (6.26)	93 (5.63)	121 (6.84)	1.00	1.23 (1.03, 1.47)	**0.023**	1.00	**1.23 (1.03, 1.48)**	**0.024**
**Online courses**									
Bangladesh (*n* = 1599)	76 (4.75)	31 (3.45)	45 (6.42)	1.00	1.14 (0.70, 1.87)	0.60	1.00	1.24 (0.75, 2.05)	0.41
India (*n* = 5595)	805 (14.39)	362 (14.11)	443 (14.62)	1.00	1.04 (0.90, 1.21)	0.58	1.00	1.03 (0.89, 1.20)	0.70
Indonesia (*n* = 812)	5 (0.62)	2 (0.68)	3 (0.58)	1.00	0.84 (0.13, 5.25)	0.85	1.00	0.83 (0.13, 5.43)	0.85
Myanmar (*n* = 386)	2 (0.52)	2 (1.22)	0 (0.00)	1.00	___	___	1.00	___	___
Philippines (*n* = 421)	18 (4.28)	7 (3.91)	11 (4.55)	1.00	1.17 (0.37, 3.74)	0.79	1.00	1.11 (0.35, 3.56)	0.86
Vietnam (*n* = 3419)	187 (5.47)	67 (4.06)	120 (6.79)	1.00	1.72 (1.27, 2.33)	**< 0.001**	1.00	**1.74 (1.28, 2.37)**	**< 0.001**
**Playing-physically**									
Bangladesh (*n* = 1599)	673 (42.05)	352 (50.21)	321 (35.75)	1.00	0.55 (0.42, 0.72)	**< 0.001**	1.00	**0.55 (0.42, 0.73)**	**< 0.001**
India (*n* = 5595)	4768 (85.22)	2303 (89.79)	2465 (81.35)	1.00	0.50 (0.40, 0.62)	**< 0.001**	1.00	**0.49 (0.39, 0.62)**	**< 0.001**
Indonesia (*n* = 812)	355 (43.72)	136 (46.58)	219 (42.12)	1.00	0.83 (0.62, 1.12)	0.23	1.00	0.83 (0.61, 1.13)	0.23
Myanmar (*n* = 386)	208 (53.89)	110 (67.07)	98 (44.14)	1.00	0.39 (0.25, 0.61)	**< 0.001**	1.00	**0.36 (0.23, 0.58)**	**< 0.001**
Philippines (*n* = 421)	205 (48.69)	105 (58.66)	100 (41.32)	1.00	0.50 (0.30, 0.82)	**0.007**	1.00	**0.49 (0.30, 0.82)**	**0.006**
Vietnam (*n* = 3419)	1736 (50.78)	984 (59.60)	752 (42.53)	1.00	0.50 (0.41, 0.61)	**< 0.001**	1.00	**0.49 (0.41, 0.60)**	**< 0.001**
**Sleeping in daytime**									
Bangladesh (*n* = 1599)	1102 (68.92)	474 (67.62)	628 (69.93)	1.00	1.11 (0.92, 1.35)	0.27	1.00	1.13 (0.93, 1.37)	0.23
India (*n* = 5595)	4311 (77.05)	1921 (74.89)	2390 (78.88)	1.00	1.25 (1.09, 1.43)	**0.001**	1.00	**1.26 (1.10, 1.43)**	**0.001**
Indonesia (*n* = 812)	214 (26.35)	81 (27.74)	133 (25.58)	1.00	0.90 (0.65, 1.24)	0.51	1.00	0.90 (0.65, 1.26)	0.55
Myanmar (*n* = 386)	194 (50.26)	67 (40.85)	127 (57.21)	1.00	1.94 (1.25, 3.01)	**0.003**	1.00	**1.95 (1.27, 3.00)**	**0.002**
Philippines (*n* = 421)	297 (70.54)	120 (67.04)	177 (73.14)	1.00	1.34 (0.90, 1.99)	0.15	1.00	1.33 (0.88, 2.02)	0.18
Vietnam (*n* = 3419)	805 (23.54)	358 (21.68)	447 (25.28)	1.00	1.22 (1.05, 1.42)	**0.010**	1.00	**1.22 (1.05, 1.42)**	**0.010**
**Watching TV**									
Bangladesh (*n* = 1599)	1124 (70.29)	484 (69.04)	640 (71.27)	1.00	1.11 (0.89, 1.40)	0.35	1.00	1.08 (0.86, 1.36)	0.52
India (*n* = 5595)	4308 (77.00)	1981 (77.23)	2327 (76.80)	1.00	0.98 (0.86, 1.11)	0.71	1.00	0.98 (0.85, 1.11)	0.72
Indonesia (*n* = 812)	298 (36.7)	106 (36.30)	192 (36.92)	1.00	1.03 (0.78, 1.35)	0.84	1.00	1.03 (0.78, 1.36)	0.82
Myanmar (*n* = 386)	229 (59.33)	90 (54.88)	139 (62.61)	1.00	1.38 (0.82, 2.31)	0.23	1.00	1.38 (0.82, 2.32)	0.23
Philippines (*n* = 421)	322 (76.48)	144 (80.45)	188 (77.69)	1.00	0.85 (0.58, 1.24)	0.39	1.00	0.83 (0.55, 1.25)	0.37
Vietnam (*n* = 3419)	1956 (57.21)	927 (56.15)	1029 (58.20)	1.00	1.09 (0.93, 1.27)	0.29	1.00	1.09 (0.93, 1.28)	0.27
**Playing games in TV, Phones, tabs, etc. (using electronic devices)**									
Bangladesh (*n* = 1599)	625 (39.09)	318 (45.36)	307 (34.19)	1.00	0.63 (0.48, 0.82)	**0.001**	1.00	**0.65 (0.50, 0.84)**	**0.001**
India (*n* = 5595)	3718 (66.45)	1801 (70.21)	1917 (63.27)	1.00	0.73 (0.65, 0.83)	**< 0.001**	1.00	**0.72 (0.63, 0.82)**	**< 0.001**
Indonesia (*n* = 812)	155 (19.09)	70 (23.97)	85 (16.35)	1.00	0.62 (0.43, 0.89)	**0.011**	1.00	**0.61 (0.42, 0.90)**	**0.013**
Myanmar (*n* = 386)	127 (32.90)	61 (37.20)	66 (29.73)	1.00	0.71 (0.46, 1.10)	0.13	1.00	0.72 (0.47, 1.10)	0.13
Philippines (*n* = 421)	240 (57.00)	104 (58.10)	136 (56.20)	1.00	0.93 (0.63, 1.36)	0.69	1.00	0.94 (0.64, 1.36)	0.73
Vietnam (*n* = 3419)	833 (24.36)	495 (29.98)	338 (19.12)	1.00	0.55 (0.47, 0.64)	**< 0.001**	1.00	**0.55 (0.47, 0.65)**	**< 0.001**

AOR models were adjusted by type of community (rural vs urban) and adolescents age (years)

#### Leisure activities

**Fig. 2:**
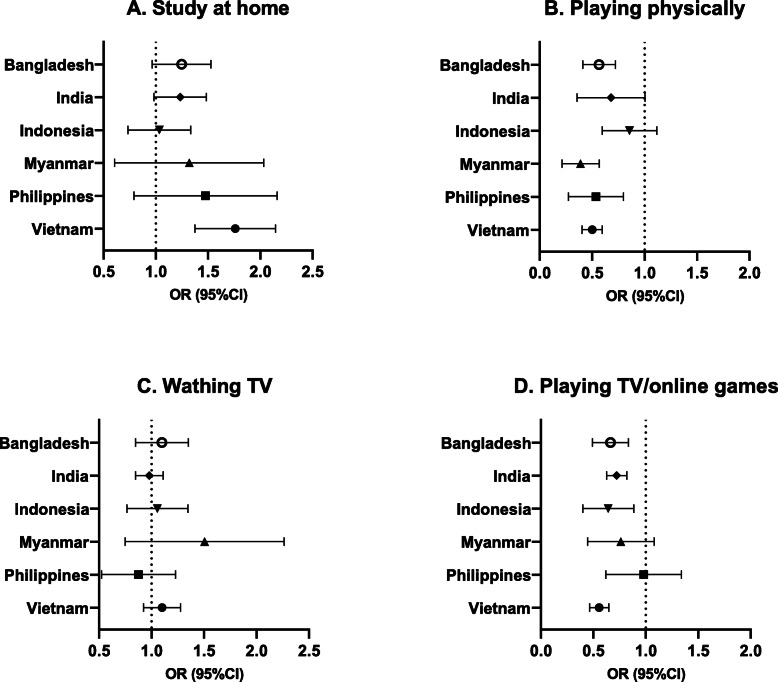
Associations between adolescent gender and daily activities in Asia Pacific countries during early COVID-19

The proportion of physical activity was moderate from 42.1 to 85.2%. Female adolescents played less physically (35.8–81.4%) than the male adolescents (46.6–89.8%) in six countries and significantly lower in the five out of these six countries (Bangladesh, AOR = 0.55, 95% CI:0.42, 0.73, *p* < 0.001; India, AOR = 0.49, 95%CI:0.39, 0.62, p < 0.001; Myanmar, AOR = 0.36, 95%CI:0.23, 0.58, p < 0.001; the Philippines, AOR = 0.49, 95%CI:0.30, 0.82, *p* = 0.006; and Vietnam, AOR = 0.49, 95%CI:0.41, 0.60, p < 0.001, respectively) (Table 2 & Fig. 2). Female adolescents had 1.26 times (95%CI:1.10, 1.43, *p* = 0.001), 1.95 times (95%CI:1.27, 3.00, *p* = 0.002), and 1.22 times (95%CI:1.05, 1.42, *p* = 0.01) higher odds of sleeping in daytime than their male counterparts in India, Myanmar, and Vietnam, respectively. The proportion of watching TV in the previous day was as high as 36.7 to 77.0% in the six countries. No significant associations were found between gender and watching TV across six countries. Playing video game was more common in male (24.0 to 70.2%) than female adolescents (16.4 to 63.3%) in six countries. Significantly lower odds were found among females in Bangladesh (AOR = 0.65, 95%CI:0.50, 0.84, *p* = 0.001), India (AOR = 0.72, 95%CI:0.63, 0.82, *p* < 0.001), Indonesia (AOR = 0.61, 95%CI:0.42, 0.90, *p* = 0.01), and Vietnam (AOR = 0.55, 95%CI:0.47, 0.65, p < 0.001) than males.

### Psychosocial status

#### Feeling isolated/stressed

**Fig. 3:**
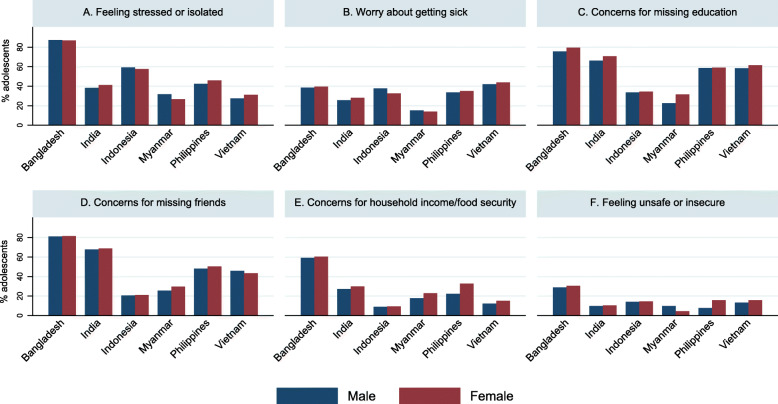
Current concerns among adolescents in Asia Pacific countries during early COVID-19

**Fig. 4:**
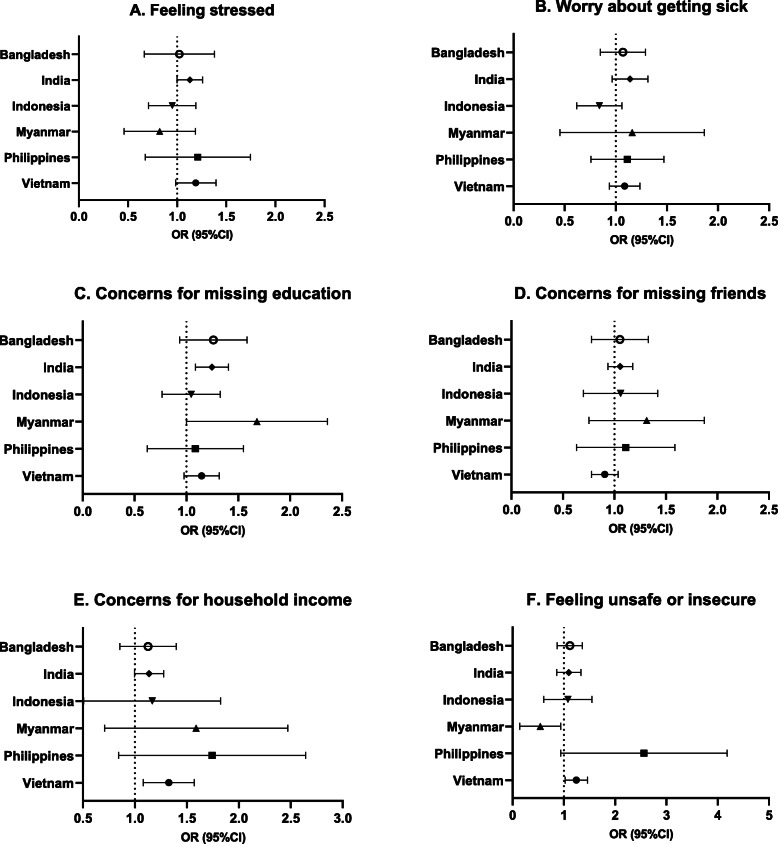
Associations between adolescent gender and current concerns in Asia Pacific countries during early COVID-19

Bangladesh had the largest percentage of both male adolescents (87.3%) and female adolescents (86.86%) who felt isolated or stressed than the remaining countries (Table 3 & Fig. 3). In India, female adolescents had 1.13 times (95%CI:1.00, 1.26, *p* = 0.047) higher odds of feeling isolated or stressed than male adolescents (Fig. 4). Although not statistically significant, female adolescents in Vietnam showed marginally a higher risk at feeling isolated than male adolescents (AOR = 1.18, 95%CI:0.99, 1.40, *p* = 0.06).

**Table 3:** Logistic regressions of adolescent gender for current concerns in Asia Pacific countries during early COVID-19

	Total	Male	Female	Male	Female	*p* value	Male	Female	*p* value
**Feeling stressed or isolated**	n (%)	n (%)	n (%)	COR	COR (95%CI)		AOR	AOR (95%CI)	
Bangladesh (*n* = 1599)	1392 (87.05)	619 (87.30)	780 (86.86)	1.00	0.96 (0.66, 1.39)	0.83	1.00	0.98 (0.69, 1.40)	0.93
India (*n* = 5595)	2227 (39.80)	981 (38.25)	1246 (41.12)	1.00	1.13 (1.00, 1.27)	0.05	1.00	**1.13 (1.00, 1.26)**	**0.047**
Indonesia (*n* = 812)	472 (58.13)	173 (59.25)	299 (57.50)	1.00	0.93 (0.73, 1.19)	0.57	1.00	0.93 (0.72, 1.20)	0.56
Myanmar (*n* = 386)	111 (28.76)	52 (31.71)	59 (26.58)	1.00	0.78 (0.50, 1.22)	0.28	1.00	0.77 (0.49, 1.21)	0.26
Philippines (*n* = 421)	187 (44.42)	76 (42.46)	111 (45.87)	1.00	1.15 (0.74, 1.79)	0.54	1.00	1.13 (0.72, 1.78)	0.59
Vietnam (*n* = 3419)	1002 (29.31)	453 (27.44)	549 (31.05)	1.00	1.19 (1.01, 1.41)	**0.038**	1.00	1.18 (0.99, 1.40)	0.06
**Worry about getting sick**									
Bangladesh (*n* = 1599)	626 (39.15)	270 (38.52)	356 (39.64)	1.00	1.05 (0.86, 1.29)	0.65	1.00	1.05 (0.86, 1.30)	0.62
India (*n* = 5595)	1505 (26.90)	656 (25.58)	849 (28.02)	1.00	1.13 (0.96, 1.33)	0.13	1.00	1.13 (0.97, 1.32)	0.13
Indonesia (*n* = 812)	280 (34.48)	110 (37.67)	170 (32.69)	1.00	0.80 (0.61, 1.05)	0.11	1.00	0.82 (0.63, 1.07)	0.15
Myanmar (*n* = 386)	56 (14.51)	25 (15.24)	31 (13.96)	1.00	0.90 (0.44, 1.86)	0.78	1.00	0.89 (0.43, 1.84)	0.75
Philippines (*n* = 421)	145 (34.44)	60 (33.52)	85 (35.12)	1.00	1.07 (0.75, 1.53)	0.70	1.00	1.07 (0.78, 1.49)	0.67
Vietnam (*n* = 3419)	1467 (42.94)	691 (41.93)	776 (43.89)	1.00	1.08 (0.94, 1.25)	0.26	1.00	1.08 (0.94, 1.24)	0.28
**Concerns for not going to school**									
Bangladesh (*n* = 1599)	1244 (77.80)	530 (75.61)	714 (79.51)	1.00	1.25 (0.97, 1.62)	0.09	1.00	1.23 (0.95, 1.60)	0.12
India (*n* = 5595)	3843 (68.69)	1699 (66.24)	2144 (70.76)	1.00	1.23 (1.08, 1.40)	**0.002**	1.00	**1.24 (1.09, 1.41)**	**0.001**
Indonesia (*n* = 812)	277 (34.11)	98 (33.56)	179 (34.42)	1.00	1.04 (0.80, 1.34)	0.77	1.00	1.02 (0.78, 1.34)	0.86
Myanmar (*n* = 386)	107 (27.72)	37 (22.56)	70 (31.53)	1.00	1.58 (1.04, 2.40)	**0.032**	1.00	**1.59 (1.05, 2.40)**	**0.028**
Philippines (*n* = 421)	248 (58.91)	105 (58.66)	143 (59.09)	1.00	1.02 (0.66, 1.57)	0.94	1.00	1.02 (0.66, 1.58)	0.94
Vietnam (*n* = 3419)	2048 (59.95)	961 (58.31)	1087 (61.48)	1.00	1.14 (0.98, 1.33)	0.09	1.00	1.14 (0.98, 1.32)	0.09
**Concerns for missing friends**									
Bangladesh (*n* = 1599)	1300 (81.30)	568 (81.03)	732 (81.51)	1.00	1.03 (0.78, 1.36)	0.82	1.00	1.03 (0.79, 1.34)	0.84
India (*n* = 5595)	3821 (68.29)	1737 (67.72)	2084 (68.78)	1.00	1.05 (0.94, 1.17)	0.39	1.00	1.05 (0.94, 1.18)	0.39
Indonesia (*n* = 812)	170 (20.94)	60 (20.55)	110 (21.15)	1.00	1.04 (0.74, 1.45)	0.83	1.00	1.02 (0.72, 1.44)	0.93
Myanmar (*n* = 386)	108 (27.98)	42 (25.61)	66 (29.73)	1.00	1.23 (0.79, 1.90)	0.36	1.00	1.23 (0.80, 1.91)	0.35
Philippines (*n* = 421)	208 (49.41)	86 (48.04)	122 (50.41)	1.00	1.10 (0.70, 1.73)	0.68	1.00	1.04 (0.67, 1.62)	0.85
Vietnam (*n* = 3419)	1521 (44.53)	755 (45.81)	766 (43.33)	1.00	0.90 (0.78, 1.04)	0.17	1.00	0.90 (0.78, 1.04)	0.16
**Concerns for household income / food security**									
Bangladesh (*n* = 1599)	957 (59.85)	414 (59.06)	543 (60.47)	1.00	1.08 (0.87, 1.34)	0.63	1.00	1.10 (0.87, 1.41)	0.42
India (*n* = 5595)	1601 (28.61)	697 (27.17)	904 (29.83)	1.00	1.14 (1.01, 1.29)	**0.040**	1.00	**1.13 (1.00, 1.28)**	**0.049**
Indonesia (*n* = 812)	75 (9.24)	26 (8.90)	49 (9.42)	1.00	1.06 (0.60, 1.89)	0.83	1.00	1.04 (0.58, 1.88)	0.90
Myanmar (*n* = 386)	80 (20.73)	29 (17.68)	51 (22.97)	1.00	1.39 (0.83, 2.34)	0.22	1.00	1.43 (0.80, 2.54)	0.23
Philippines (*n* = 421)	119 (28.27)	40 (22.35)	79 (32.64)	1.00	1.68 (0.99, 2.87)	0.06	1.00	1.59 (0.93, 2.71)	0.09
Vietnam (*n* = 3419)	469 (13.73)	201 (12.20)	268 (15.16)	1.00	1.29 (1.08, 1.53)	**0.005**	1.00	**1.31 (1.09, 1.58)**	**0.005**
**Feeling unsafe or insecure**									
Bangladesh (*n* = 1599)	475 (29.71)	202 (28.82)	273 (30.40)	1.00	1.08 (0.87, 1.34)	0.50	1.00	1.10 (0.88, 1.37)	0.40
India (*n* = 5595)	566 (10.12)	249 (9.71)	317 (10.46)	1.00	1.09 (0.87, 1.35)	0.46	1.00	1.08 (0.87, 1.34)	0.49
Indonesia (*n* = 812)	116 (14.29)	41 (14.04)	75 (14.42)	1.00	1.03 (0.65, 1.63)	0.89	1.00	1.01 (0.65, 1.58)	0.95
Myanmar (*n* = 386)	26 (6.74)	16 (9.76)	10 (4.50)	1.00	0.44 (0.20, 0.97)	**0.042**	1.00	**0.44 (0.20, 0.98)**	**0.044**
Philippines (*n* = 421)	52 (12.35)	14 (7.82)	38 (15.70)	1.00	2.20 (1.12, 4.29)	**0.021**	1.00	**2.22 (1.14, 4.33)**	**0.019**
Vietnam (*n* = 3419)	495 (14.49)	217 (13.17)	278 (15.72)	1.00	1.23 (1.03, 1.47)	**0.021**	1.00	**1.23 (1.03, 1.47)**	**0.022**

AOR models were adjusted by type of community (rural vs. urban) and adolescents age (years)

#### Worrying about getting sick

The proportion of respondents worrying about getting sick ranged from 14.5% in Myanmar to 42.9% in Vietnam. Across the six countries, there was no significant difference between female and male adolescents in worrying about getting sick (Table 3, Figs. 3 & 4).

#### Concerns for not going to school

In Bangladesh, India, the Philippines, and Vietnam, more than 50% of adolescents (both males and females) reported concerns for not going to school. Female adolescents in India and Myanmar had 1.24 times (95%CI:1.09, 1.41, *p* = 0.001) and 1.59 times (95%CI:1.05, 2.40, *p* = 0.03) higher odds of concerning for not going to school compared to male adolescents (Table 3, Figs. 3 & 4).

#### Concerns for missing friends

In India and Bangladesh, 68.3 and 81.3% of respondents, respectively, presented concerns for missing friends, which was relatively higher than the other countries. No significant relationships between gender and concerns for missing friends were found across these six countries (Table 3, Figs. 3 & 4).

#### Concerns for household income or food security

About 60% Bangladesh adolescents expressed the concern of household income or food security, which was higher than other five countries. Female adolescents had higher odds compared to male adolescents in India (AOR = 1.13; 95%CI:1.00, 1.28, *p* = 0.049) and Vietnam (AOR = 1.31; 95%CI:1.09, 1.58, *p* = 0.005), while other countries did not show significant gender difference (Table 3, Figs. 3 & 4).

#### Feeling unsafe or insecure

The findings of feeling unsafe or insecure is mixed among countries. Female adolescents in the Philippines and Vietnam had 2.22 times (95%CI:1.14, 4.33, *p* = 0.02) and 1.23 times (95%CI:1.03, 1.47, p = 0.02) higher odds about feeling unsafe or insecure than their male counterparts. However, female adolescents had lower odds (AOR = 0.44; 95%CI:0.20, 0.98, *p* = 0.04) of feeling unsafe, compared to male adolescents in Myanmar (Table 3, Figs. 3 & 4).

### Information access related to the COVID-19

#### Television

Generally, television was the most common source of COVID-19 information among respondents (74.1–88.1%) in all countries (Table 4). There was no significant difference in watching TV by adolescent gender across the six countries.

**Table 4:** Logistic regressions of adolescent gender for COVID-19 information sources in Asia Pacific countries

	Total	Male	Female	Male	Female	*p* value	Male	Female	*p* value
	n (%)	n (%)	n (%)	COR	COR (95%CI)		AOR	AOR (95%CI)	
**Television**									
Bangladesh (*n* = 1599)	1408 (88.06)	610 (87.02)	798 (88.86)	1.00	1.19 (0.85, 1.66)	0.30	1.00	1.17 (0.84, 1.62)	0.35
India (*n* = 5595)	4401 (78.77)	2012 (78.44)	2395 (79.04)	1.00	1.04 (0.91, 1.18)	0.58	1.00	1.03 (0.91, 1.18)	0.61
Indonesia (*n* = 812)	601 (74.01)	222 (76.03)	379 (72.88)	1.00	0.85 (0.62, 1.17)	0.31	1.00	0.84 (0.61, 1.16)	0.29
Myanmar (*n* = 386)	290 (75.13)	120 (73.17)	170 (76.58)	1.00	1.20 (0.75, 1.91)	0.45	1.00	1.22 (0.76, 1.97)	0.41
Philippines (*n* = 421)	362 (85.99)	156 (87.15)	206 (85.12)	1.00	0.84 (0.46, 1.53)	0.58	1.00	0.83 (0.45, 1.54)	0.55
Vietnam (*n* = 3419)	2556 (74.82)	1209 (73.36)	1347 (76.19)	1.00	1.16 (0.95, 1.42)	0.14	1.00	1.17 (0.95, 1.43)	0.13
**Internet/social media**									
Bangladesh (*n* = 1599)	751 (46.97)	396 (56.49)	355 (39.53)	1.00	0.50 (0.40, 0.64)	**< 0.001**	1.00	**0.51 (0.41, 0.64)**	**< 0.001**
India (*n* = 5595)	2342 (41.86)	1127 (43.94)	1215 (40.10)	1.00	0.85 (0.75, 0.97)	**0.019**	1.00	**0.84 (0.73, 0.96)**	**0.010**
Indonesia (*n* = 812)	292 (35.96)	103 (35.27)	189 (36.35)	1.00	1.05 (0.74, 1.49)	0.80	1.00	1.07 (0.76, 1.51)	0.70
Myanmar (*n* = 386)	107 (27.72)	54 (32.93)	53 (23.87)	1.00	0.64 (0.42, 0.97)	**0.034**	1.00	**0.65 (0.43, 0.97)**	**0.036**
Philippines (*n* = 421)	238 (56.53)	86 (48.04)	152 (62.81)	1.00	1.83 (1.30, 2.58)	**0.001**	1.00	**1.85 (1.27, 2.70)**	**0.001**
Vietnam (*n* = 3419)	2307 (67.54)	1106 (67.11)	1201 (67.93)	1.00	1.04 (0.87, 1.24)	0.68	1.00	1.06 (0.89, 1.27)	0.50
**Mobile (phone call/SMS)**									
Bangladesh (*n* = 1599)	947 (59.22)	407 (58.06)	540 (60.13)	1.00	1.09 (0.85, 1.39)	0.49	1.00	1.12 (0.88, 1.43)	0.35
India (*n* = 5595)	3581 (64.00)	1677 (65.38)	1904 (62.84)	1.00	0.90 (0.82, 0.98)	**0.020**	1.00	**0.88 (0.80, 0.98)**	**0.014**
Indonesia (*n* = 812)	132 (16.26)	51 (17.47)	81 (15.58)	1.00	0.87 (0.58, 1.31)	0.51	1.00	0.83 (0.56, 1.23)	0.35
Myanmar (*n* = 386)	50 (12.95)	24 (14.63)	26 (11.71)	1.00	0.77 (0.46, 1.29)	0.33	1.00	0.79 (0.48, 1.29)	0.35
Philippines (*n* = 421)	70 (16.63)	25 (13.97)	45 (18.60)	1.00	1.41 (0.79, 2.49)	0.24	1.00	1.40 (0.77, 2.55)	0.28
Vietnam (*n* = 3419)	1797 (52.61)	839 (50.91)	958 (54.19)	1.00	1.14 (0.98, 1.33)	0.10	1.00	1.15 (0.98, 1.35)	0.08
**Friends**									
Bangladesh (*n* = 1599)	665 (41.59)	300 (42.80)	365 (40.65)	1.00	0.92 (0.76, 1.10)	0.35	1.00	0.96 (0.80, 1.15)	0.63
India (*n* = 5595)	2991 (53.46)	1385 (54.00)	1606 (53.00)	1.00	0.96 (0.86, 1.07)	0.47	1.00	0.96 (0.86, 1.06)	0.41
Indonesia (*n* = 812)	181 (22.29)	64 (21.92)	117 (22.50)	1.00	1.03 (0.75, 1.42)	0.84	1.00	1.07 (0.78, 1.47)	0.67
Myanmar (*n* = 386)	98 (25.39)	39 (23.78)	59 (26.58)	1.00	1.16 (0.70, 1.91)	0.56	1.00	1.17 (0.71, 1.94)	0.54
Philippines (*n* = 421)	42 (9.98)	22 (12.29)	20 (8.26)	1.00	0.64 (0.30, 1.36)	0.25	1.00	0.56 (0.26, 1.22)	0.14
Vietnam (*n* = 3419)	1749 (51.20)	809 (49.09)	940 (53.17)	1.00	1.18 (1.02, 1.36)	**0.030**	1.00	**1.18 (1.02, 1.36)**	**0.029**
**Family**									
Bangladesh (*n* = 1599)	945 (59.10)	377 (53.78)	568 (63.25)	1.00	1.48 (1.24, 1.77)	**< 0.001**	1.00	**1.44 (1.21, 1.70)**	**< 0.001**
India (*n* = 5595)	3439 (61.47)	1495 (58.28)	1944 (64.16)	1.00	1.28 (1.14, 1.43)	**< 0.001**	1.00	**1.29 (1.15, 1.45)**	**< 0.001**
Indonesia (*n* = 812)	305 (37.56)	106 (36.30)	199 (38.27)	1.00	1.09 (0.78, 1.51)	0.62	1.00	1.07 (0.77, 1.50)	0.67
Myanmar (*n* = 386)	164 (42.49)	70 (42.68)	94 (42.34)	1.00	0.99 (0.65, 1.49)	0.95	1.00	0.97 (0.64, 1.47)	0.89
Philippines (*n* = 421)	115 (27.32)	56 (31.28)	59 (24.38)	1.00	0.71 (0.42, 1.21)	0.20	1.00	0.72 (0.42, 1.24)	0.23
Vietnam (*n* = 3419)	1989 (58.23)	947 (57.46)	1042 (58.94)	1.00	1.06 (0.93, 1.21)	0.36	1.00	1.06 (0.93, 1.21)	0.38

AOR models were adjusted by type of community (rural vs. urban) and adolescents age (years)

#### Internet/social media

Relatively smaller percentages of adolescents in Indonesia and Myanmar got such information through the internet (35.96 and 27.7%, respectively) compared to other countries (41.9–67.5%). Female adolescents were less likely to get information from the internet or social media than male adolescents in Bangladesh (AOR = 0.51, 95%CI:0.41, 0.64, *p* < 0.001), India (AOR = 0.84, 95%CI:0.73, 0.96, *p* = 0.01), and Myanmar (AOR = 0.65, 95%CI:0.43, 0.97, p = 0.04). But in the Philippines, female adolescents had 1.85 times (95%CI:1.27, 2.70, *p* = 0.001) higher odds of receiving COVID-19 information from the internet than male counterparts.

#### Phone call / SMS

The percentages of using phone call or short massage to receive COVID-19 information were only 16.3, 13.0 and 16.6% in Indonesia, Myanmar, and the Philippines respectively. In India, female adolescents had lower odds (AOR = 0.88; 95%CI:0.80, 0.98, *p* = 0.01) of receiving COVID-19 information through mobile sources than male adolescents. No significant difference by gender in receiving information via phone call or massages were found in other five countries.

#### Friends

Friends (10.0–53.5%) was a major channel to get COVID-19 related information. For utilizing friends as information sources, female participants had 1.18 times (95%CI:1.02, 1.36, *p* = 0.03) higher odds than males in Vietnam. The patterns were mixed and not significant among other five countries.

#### Family

Family was mentioned with a range of 27.3–61.5% in six countries Female adolescents were 1.44 times (95%CI:1.21, 1.70, *p* < 0.001) and 1.29 times (95%CI:1.15, 1.45, p < 0.001) more likely to receive information from their family, compared to male adolescents in Bangladesh and India.

## Discussion

To our knowledge, this study was the first multi-country study to investigate the differences in psychosocial status among male and female adolescents impacted by the COVID-19 pandemic in the Asia Pacific region. This study provides evidence for response and recovery strategies from COVID-19 by analyzing adolescents’ daily activities, stressors, and information acquisition channels. Findings suggested that COVID-19 lockdown measures had different impacts on adolescent mental health or psychosocial status by gender in these six countries, but with some variations. In Bangladesh and Indonesia, higher percentages of adolescents felt stressed and isolated by COVID-19 than in other countries. Female adolescents were more likely to have concerns related to the COVID-19 pandemic than males. Gender differences were also found in daily activities. Male adolescents were more likely to report engaging in physical activities and video games than females in general. In terms of study activities, online or remote education was accessed by only limited percentages of adolescents. With regard to information acquisition, female adolescents were less likely than males to use internet/mobile phones to get COVID-19 related information, while they were more likely to use friends or family as information channels.

### Lack of access to distance learning across countries

The present study showed strikingly low proportions of access to remote education or online courses across six countries, suggesting poor distance learning systems in these countries and poor access to these systems for vulnerable youths. Adolescents in resource-poor countries would be the most vulnerable learners during this global shock, due to a lack of accessibility to school materials and instruction required for distance learning during school closures [30]. Even before the pandemic, countries in the Asia Pacific Region were struggling with inequity of education issues [50]. From the beginning of lockdown measures in early 2020 until January 2021, the surveyed countries had experienced educational disruptions for more than one-third academic year on average: Bangladesh with 38 weeks, India with 47 weeks, Indonesia with 39 weeks, Myanmar with 39 weeks, the Philippines with 27 weeks, and Vietnam with 12 weeks [49]. The disruption of education can jeopardize not only adolescent’s education and academic progress, but also physical safety and mental health in the short- and long-term. If there is not rapid and effective remediation, the loss of learning in these foundational years will increase the learning gaps in subsequent years, and increase drop-out rates [51]. The school closures, along with the pandemic’s economic impacts, might affect parents’ decisions regarding their child’s education, and could potentially cost children the opportunity to complete their education, subsequently deepening the education equity gaps that existed before the pandemic [52]. In some Asian countries, male children are considered first for educational opportunities compared to their female siblings [32, 33], which may leave school-aged females with even more reduced access to education and higher drop-out rates during and after the pandemic compared to male counterparts. Dropping out of school may also increase the rate of child marriage and early pregnancies [53].

Our findings showed significant gender differences in study activities only in Vietnam, with female adolescents having higher odds ratio for studying at home than males. This may be attributed to the efforts of Vietnamese government to improve gender equity in education in recent decades. However, even as the education gender gap has begun to close for primary education in Vietnam, challenges still remain, especially for minority groups, and in secondary and post-secondary education [54]. Taken together, in addition to considering how to provide adequate and appropriate education for all school-aged adolescents during and after pandemic, policy makers need to eliminate the gender inequity that may be enlarged by the education disruption.

### Lack of physical activity among female students

Staying-at-home has increased sedentary behavior, limited access to public services, and decreased physical activity for both female and male youth, which has been associated with a higher risk of impeding adolescent mental and physical health and social-emotional development [30, 55, 56]. The present study revealed a lack of physical activities among female adolescents relative to males in five countries except for Indonesia. Our findings on physical activity supported previous studies before the COVID-19 pandemic began which showed that adolescent girls participate less in physical activities than their male counterparts in countries like India and Bangladesh [57, 58]. The reasons for these gender differences could be that male adolescents demonstrated greater self-efficacy in overcoming barriers to physical activities and higher levels of participation in community physical activities [59]. Moreover, in India, the barriers for female youth also included negative body image and social censure by participating in physical activities [57]. In many Asian countries, it is more culturally and socially acceptable for boys to participate in formal organized sports and group physical activities than for girls [57].

### Higher vulnerability to psychosocial distress among females

Findings in the current study indicated that large proportions of respondents felt boredom or missed friends during the lockdown. Schools are the primary social venues for school-aged adolescents; thus, the closure of schools has cut off channels for interaction, and thrust them into social isolation [60], which could have long-term negative effects on mental health [61]. Apart from the school closures, other collateral effects that come with the lockdown may also influence adolescent psychosocial status and result in a high rates of COVID-19 related stress among the respondents in this study. Previous studies showed that having relatives or neighbors who had tested positive for diseases, like the H1N1 or COVID-19, may increase fears of illness and death among children [62, 63].

The present study showed that females are more vulnerable under stress caused by COVID-19. Previous evidence reported that, globally, female adolescents were more likely to have negative emotions and higher levels of psychological distress than their male counterparts during the COVID-19 pandemic [13, 20, 64–68]. Gender differences, such as higher sensitivity to traumatic events, may contribute to the higher level and prevalence of psychological morbidity among females than males [69]. Higher level of stress faced by female adolescents than their male peers in Asia Pacific countries may also be attributed to socio-economic stressors. For example, past evidence from India suggested that girls are more likely to be pushed into the labor market to compensate for economic loss of the family caused by COVID-19 restrictions [35]. In addition, the restrictions increased the risk of domestic or sexual abuse in South Asia among young females, especially those in extreme poverty [70].

Given the negative impacts brought by the restriction measures and COVID-19 pandemic itself, it calls for timely interventions to address adolescents’ psychosocial needs. Psychosocial distress experienced by adolescents, if abandoned, will also impede their physical and mental health in their adulthood [27]. Policy makers and social organizations need to aware that female adolescents may face more complicated situations, while developing and implementing such interventions in the region.

### Different information resources for COVID-19 by gender

Accessing to COVID-19 information is important for adolescents to get adequate knowledge of COVID-19. Such knowledge was proved to be significantly associated with COVID-19 related risk perception and practicing protective behaviors among dental students in India [28, 71]. In our study, female adolescents were more likely to get COVID-19- related information from family or friends and had less access to the internet or mobile phones, compared to male adolescents. This gender disparity could be explained by existing gender disparities in accessing to mobile and internet in these countries. According to the Mobile Gender Gap Report [72], Bangladesh had the highest gender gap in Asian countries surveyed, where 58% of adult women were less likely to use mobile internet than males, followed by India (56%), Myanmar (39%), and Indonesia (18%), respectively. Evidence showed that the Philippines made great progress in making the internet accessible to females [73], which may contribute to the outcome that the female adolescents were more likely to have COVID-19 information via internet than males in the Philippines. Getting access to internet could increase adolescents’ perceptions of inclusiveness [71], thereby it has salient implications to adolescents’ mental health during COVID-19. By and large, due to less access to open information sources (i.e., internet), female adolescents may get less exposure to means that could support their physical and mental health.

### Implications

Evidence-based response and recovery interventions are a top necessity to help reduce adverse psychosocial impacts on adolescents. This study demonstrates that female adolescents have different psychosocial needs than their male peers, shows how these needs may have changed due to the pandemic, and how these needs depend on culture and context. Our findings have implications for social support, policy making by governments, and planning post-pandemic response and recovery strategies by organizations, like UNICEF.

First, alternative academic supports are urgently needed to improve education opportunities and life quality for affected adolescents from low-income Asian countries. It is a challenge to prepare facilities and techniques for remote education on a large scale in a short period of time. Thus, governments of the surveyed countries have already taken measures to compensate K-12 education service. For instance, Bangladesh applied a multi-modal approach including television, radio, internet, and mobile phones [74]. A survey conducted in the Philippines showed only 64% students are willing to engage in distance learning via computer or other digital devices [75]. Hence, the coverage rate and effectiveness of such models still need to be monitored and evaluated, especially in rural areas.

Second, psychosocial interventions for adolescents should extend to the community and family, considering the local cultural characteristics, focusing on gender difference. For example, nationwide community healthcare networks, such as Bangladesh Rural Advancement Committee (BRAC) in Bangladesh, could be utilized to deliver services to the remotest areas [76]. As most people in the Asia Pacific region are religious, understanding local religious culture and cooperating with local health workers or faith leaders would help policy makers and social organizations to better identify the psychosocial needs of adolescents. In addition, with the increasing challenges faced by families, parents’ emotional resources are drained, and their parenting capacity is reduced [77]. Plus, the level of social support was suggested to be inversely associated with prevalence of depression and anxiety symptoms among adolescents [65], with parental support being the most important source of social support in adolescent life [78]. However, such evidence is lacking among the surveyed six countries.

Third, beyond academic and psychosocial support at a general population level, the recovery efforts from policy makers and social organizations should provide more specific and safety COVID-related information services for adolescents in Asia Pacific countries. As mentioned previously, information accuracy and transparency will reduce the uncertainty and fear of the virus and lockdown policies. It is important to analyze the sources where adolescents frequently seek information to understand how we can disseminate sufficient and effective information to them. Our findings suggested that the channels of receiving COVID-19 related information differ by child gender. This suggest that the COVID-19 information channels need to be diversified considering gender sensitivity. Public health organizations may have a larger presence on television outlets versus social media [79]. Actively utilizing mass media channels like TV, social media, and mobile-phone texts could be part of future strategies for rapid knowledge dissemination related to COVID-19. More attention should be paid to female adolescents to mitigate the gender disparities brought by information bias.

Fourth, follow-up investigations and strategies should be developed considering the possibilities of increased incidence of Post-Traumatic Stress Disorder (PTSD) or Post-Traumatic Stress Symptoms (PTSS) in the ongoing and post-pandemic phases. PTSD and PTSS have been reported among adolescents after traumatic events like the Gorkha earthquake in Nepal [80] and the Ebola and SARS outbreaks [81, 82]. An increased prevalence of PTSS, as a result of the COVID-19 pandemic, was already found among residents [83] and youth [84] in the hardest-hit areas in China. The limited literature in the region has identified clear gaps between the mental health services available and the demand [63, 85, 86]. Thus, to cope with a potential rise in PTSS among adolescents during and after the pandemic, recovery strategies need to be analyzed by public health organizations to ensure that they include interventions and mechanisms to help adolescents deal with PTSS.

### Limitations

There are several limitations to the present study. First, the sampling strategy varied in its detail by country. This can limit the representativeness of vulnerable adolescents in the World Vision’s program areas. The structure of the survey sampling frames varied by country and the regional aggregated data were not weighted to the survey population. Another limitation is non-probabilistic sampling, as most of the data collection occurred in areas supported by long-term development or COVID-19 relief support agencies. This selective choice of survey participants does not guarantee the generalizability of the findings.

Second, the child survey questionnaire had no information on the parent’s or caregiver’s occupation, education level, and other socioeconomic indicators. These are potential confounders related to our outcome variables. Thus, the regression analysis only accounted for rural and urban residence and child age.

Third, this study employed a self-report survey, which is more likely to introduce response bias. It would be more comprehensive to conduct analysis by integrating perceptions from both adolescents and parents in future research.

Fourth, the cross-sectional survey does not provide information prior to COVID-19 pandemic. Findings regarding lack of access to education or physical activities are therefore not solely due to the COVID-19 pandemic, but instead could be mixed with existing gaps before COVID-19.

Thus, it is also necessary to conduct a prospective longitudinal study to look deeper at the predictive indicators of adolescent psychosocial status within the context of COVID-19 over time, as the pandemic and subsequent recovery period progress.

Fifth, the surveyed adolescents were receiving different levels of assistance from World Vision and possibly other organizations. Also, the survey was conducted by World Vision staff or community members, which could introduce desirability bias among respondents.

## Conclusion

During the early stages of the COVID-19 pandemic, adolescents in the Asia Pacific region are considered at a high risk in terms of receiving education and facing gender inequity. It is important to understand their current situation to support policy makers and associated organizations come up with response and recovery actions to mitigate the negative impacts caused by the restrictions. Our findings revealed that surveyed adolescents in six countries in the Asia Pacific region were experiencing a severe disruption of education and lack of access to distance learning. During the first year of the pandemic, the physical and psychosocial status of female youth were more negatively affected than male peers. Considering the possibilities of posttraumatic symptoms, longitudinal research is needed to understand the long-term impact of COVID-19 on psycho-social status among adolescents in the region. In addition, future research could investigate support to parents on parenting, home-schooling, and mental health that would bolster parents’ well-being so as to ultimately mitigate adolescents’ psychological distress. Alongside, public health services need to be prepared for long-term mental health supports, particularly for female adolescents. Also, public health services should extend social support to community and family levels, and integrate mass media in information dissemination.

## Supplementary Information

**Additional file 1.**

## Data Availability

The data that support the findings of this study are available from World Vision, but restrictions apply to the availability of these data, which were used under license for the current study, and so are not publicly available. Data are however available from the authors upon reasonable request and with permission of World Vision.

## Abbreviations

AOR: Adjusted Odds Ratio
AP: Area Programmes
BRAC: Bangladesh Rural Advancement Committee
CI: Confidence Intervals
OR: Odds Ratio
PTSD: Post-Traumatic Stress Disorder
PTSS: Post-Traumatic Stress Symptoms
SARS: Severe Acute Respiratory Syndrome
TV: Television
UN: United Nations
UNESCO: United Nations Educational, Scientific and Cultural Organization
US: United States
WV: World Vision
