# Supplementary material for: Gender differences in psychosocial status of adolescents during COVID-19: a six-country cross-sectional survey in Asia Pacific
Source: BMC Public Health. 2021 Nov 4;21:2009. doi: 10.1186/s12889-021-12098-5 (PMC8568363; doi:10.1186/s12889-021-12098-5)
Supplement: Supplementary file 1 — Additional file 1. [file 12889_2021_12098_MOESM1_ESM.docx]

**Supplemental Table 1. Lockdown measures and distant learning situation in Asia Pacific countries during early Covid-19**

| Countries | Lockdown Measures | Distant Learning Situation | Reference |
| --- | --- | --- | --- |
| Bangladesh | Strict | Country-wide closed. 60% of households had access to internet; public broadcaster was utilized to broadcast educational context. | Chik, J. (2020). Coronavirus: What are the lockdown measures and travel bans in Asia. *South China Morning Post*. https://www.scmp.com/news/china/society/article/3078552/coronavirus-what-are-lockdown-measures-and-travel-bans-asia  UNESCO. *COVID-19 Educational Disruption and Response in Asia-Pacific.* https://apa.sdg4education2030.org/covid19  Menon, S. (2020). Coronavirus: How the lockdown has changed schooling in South Asia. *BBC,* https://www.bbc.com/news/world-south-asia-54009306 |
| India | Various in different areas | Country-wide closed. 24% of households had access to internet; only 4% in rural area; public broadcaster was utilized to broadcast educational context. | UNESCO. *COVID-19 Educational Disruption and Response in Asia-Pacific.* https://apa.sdg4education2030.org/covid19  Menon, S. (2020). Coronavirus: How the lockdown has changed schooling in South Asia. *BBC,* https://www.bbc.com/news/world-south-asia-54009306 |
| Indonesia | Less strict | Country-wide closed. 40% of population had access to internet, most in urban areas; public broadcaster was utilized to broadcast educational context. | Lee, Y. (2020). The pandemic’s effect on education is not being discussed enough, says Indonesian minister. *CNBC,*  [*https://www.cnbc.com/2020/09/14/indonesias-education-minister-on-covids-effect-on-students-learning.html*](https://www.cnbc.com/2020/09/14/indonesias-education-minister-on-covids-effect-on-students-learning.html)  UNESCO. *COVID-19 Educational Disruption and Response in Asia-Pacific.* <https://apa.sdg4education2030.org/covid19>  CSIS (2020). *Southeast Asia Covid-19 Tracker.* https:// www.csis.org/programs/southeast-asia-program/southeast-asia-covid-19-tracker-0 |
| Myanmar | Less strict; Curfews in several regions; a supplementary stay-at-home order in seven townships | Country-wide closed. School reopened in July, but closed again in August | CSIS (2020). *Southeast Asia Covid-19 Tracker.* https:// www.csis.org/programs/southeast-asia-program/southeast-asia-covid-19-tracker-0  UNESCO. *COVID-19 Educational Disruption and Response in Asia-Pacific.* https://apa.sdg4education2030.org/covid19  UN, Myanmar. (2020). Back to school after COVID-19: Safe reopening of schools. *United Nations, Myanmar,* https://myanmar.un.org/en/92061-responding-education-crisis  UN News. (2020). Learning Lessons from COVID-19 in Myanmar. *UN News,* https://news.un.org/en/story/2020/10/1074602 |
| The Philippines | Strict; shutdown from mid-March to May; stay-at-home orders in August | Country-wide closed. | BBC. (2020). Coronavirus: Millions return to lockdown in Philippines. *BBC,* <https://www.bbc.com/news/world-asia-53646149>  UNESCO. *COVID-19 Educational Disruption and Response in Asia-Pacific.* https://apa.sdg4education2030.org/covid19  CSIS (2020). *Southeast Asia Covid-19 Tracker.* https:// www.csis.org/programs/southeast-asia-program/southeast-asia-covid-19-tracker-0  Chik, H. (2020). Coronavirus: What are the lockdown measures and travel bans in Asia. *South China Morning Post*. https://www.scmp.com/news/china/society/article/3078552/coronavirus-what-are-lockdown-measures-and-travel-bans-asia |
| Vietnam | Strict in the early stage | Fully open. School closed at the end of January and reopened in May.  Social distancing period – April 1 to June 9 | UNESCO. *COVID-19 Educational Disruption and Response in Asia-Pacific.* https://apa.sdg4education2030.org/covid19  CSIS (2020). *Southeast Asia Covid-19 Tracker.* https:// www.csis.org/programs/southeast-asia-program/southeast-asia-covid-19-tracker-0  France-Presse, A. (2020). Vietnam kids back at school after 3-month virus break. *The Jakarta Post,* https://www.thejakartapost.com/seasia/2020/05/04/vietnam-kids-back-at-school-after-3-month-virus-break.html  Jones, A. (2020). Coronavirus: How ‘overreaction’ made Vietnam a virus success. *BBC,* <https://www.bbc.com/news/world-asia-52628283> |

**Supplemental Table 2. Daily activities among adolescents six Asia Pacific countries during early Covid-19**

|  | Bangladesh  (n=1599) | India  (n=5595) | Indonesia  (n=812) | Myanmar  (n=386) | Philippines  (n=421) | Vietnam  (n=3419) |
| --- | --- | --- | --- | --- | --- | --- |
|  | n (%) | n (%) | n (%) | n (%) | n (%) | n (%) |
| **Studying** | 1206 (57.42) | 4815 (86.06) | 496 (61.08) | 64 (16.58) | 144 (34.20) | 2655 (77.65) |
| Mean (SD)/min, max | 1.48 (1.39) / 0, 15 | 1.8 (1.3) / 0, 10 | / | 0.22 (0.61) / 0, 4 | 0.48 (0.82) / 0, 5 | / |
| **Remote education by school** | 226 (14.13) | 1160 (20.73) | 87 (10.71) | 2 (0.52) | 32 (7.60) | 214 (6.26) |
| Mean (SD)/min, max | 0.1 (0.3) / 0, 3 | 0.3 (0.8) / 0, 8 | / | 0.01 (0.07) / 0, 1 | 0.10 (0.41) / 0, 3 | / |
| **Online courses** | 76 (4.75) | 805 (14.39) | 5 (0.62) | 2 (0.52) | 18 (4.28) | 187 (5.47) |
| Mean (SD)/min, max | 0.03 (0.2) / 0, 2 | 0.2 (0.7) / 0, 8 | / | 0.01 (0.72) / 0, 1 | 0.10 (0.69) / 0, 12 | / |
| **Playing-physically** | 673 (42.05) | 4768 (85.22) | 355 (43.72) | 208 (53.89) | 205 (48.69) | 1736 (50.78) |
| Mean (SD)/min, max | 0.38 (0.64) / 0, 5 | 1.8 (1.3) / 0, 10 | / | 0.95 (1.36) / 0, 10.5 | 1.02 (1.62) / 0, 20 | / |
| **Sleeping in daytime** | 1102 (68.92) | 4311 (77.05) | 214 (26.35) | 194 (50.26) | 297 (70.54) | 805 (23.54) |
| Mean (SD)/min, max | 0.93 (0.92) / 0, 6 | 1.9 (2.0) / 0, 14 | / | 0.81 (1.02) / 0, 7 | 1.71 (1.62) / 0, 10 | / |
| **Watching TV** | 1124 (70.29) | 4308 (77.00) | 298 (36.70) | 229 (59.33) | 322 (76.48) | 1956 (57.21) |
| Mean (SD)/min, max | 0.91 (0.97) / 0, 7 | 1.7 (1.3) / 0, 12 | / | 1.07 (1.21) / 0, 6 | 1.90 (1.68) / 0, 12 | / |
| **Playing games in TV, Phones, tabs, etc. (using electronic devices)** | 625 (39.09) | 3718 (66.45) | 155 (19.09) | 127 (32.90) | 240 (57.00) | 833 (24.36) |
| Mean (SD)/min, max | 0.37 (0.68) / 0, 7.5 | 1.1 (1.2) / 0, 10 |  | 0.52 (1.13) / 0, 10 | 1.47 (2.2) / 0, 16 |  |

**Supplemental Table 3. Other important information sources related to the COVID-19**

|  | Indonesia  (n=812) | India  (n=5595) | Bangladesh  (n=1599) | Myanmar  (n=386) | Philippines  (n=421) | Vietnam  (n=3416) |
| --- | --- | --- | --- | --- | --- | --- |
|  | n (%) | n (%) | n (%) | n (%) | n (%) | n (%) |
| Community people/NGO | 36 (4.43) * | 251 (4.49) * | 757 (47.34) | 153 (39.64) | N/A | 86 (2.52) * |
| Radio | 87 (10.71) | 437 (7.81) | 112 (7.00) | N/A | 144 (34.20) | 633 (18.53) |

Numbers with * were collected manually from specified answers. Data collected in Indonesia and Vietnam are translated from local language.

**Supplement Table 4. Daily activities by gender among adolescents in Asia Pacific countries during early COVID-19**

|  | Total | Male | Female | *P*-value |
| --- | --- | --- | --- | --- |
|  |  | N=5552 | N=6680 |  |
| **Studying at home** | N (%) | n (%) | n (%) |  |
| Bangladesh  (n=1599) | 1206 (75.42) | 512 (73.04) | 694 (77.28) | 0.04 |
| India  (n=5595) | 4815 (86.06) | 2175 (84.80) | 2640 (87.13) | **<0.001** |
| Indonesia  (n=812) | 496 (61.08) | 178 (60.96) | 318 (61.15) | 0.96 |
| Myanmar  (n=386) | 64 (16.58) | 25 (15.24) | 39 (17.57) | 0.26 |
| Philippines  (n=421) | 144 (34.20) | 55 (30.73) | 89 (36.78) | 0.17 |
| Vietnam  (n=3419) | 2655 (77.65) | 1201 (72.74) | 1454 (82.24) | **<0.001** |
|  |  |  |  |  |
| **Remote education by school** |  |  |  |  |
| Bangladesh  (n=1599) | 226 (14.13) | 87 (12.41) | 139 (15.48) | **0.035** |
| India  (n=5595) | 1,160 (20.73) | 540 (21.05) | 620 (20.46) | 0.98 |
| Indonesia  (n=812) | 87 (10.71) | 37 (12.67) | 50 (9.62) | 0.18 |
| Myanmar  (n=386) | 2 (0.52) | 1 (0.61) | 1 (0.45) | 0.83 |
| Philippines  (n=421) | 32 (7.60) | 8 (4.47) | 24 (9.92) | 0.08 |
| Vietnam  (n=3419) | 214 (6.26) | 93 (5.63) | 121 (6.84) | 0.14 |
|  |  |  |  |  |
| **Online courses** |  |  |  |  |
| Bangladesh  (n=1599) | 76 (4.75) | 31 (3.45) | 45 (6.42) | 0.50 |
| India  (n=5595) | 805 (14.39) | 362 (14.11) | 443 (14.62) | 0.58 |
| Indonesia  (n=812) | 5 (0.62) | 2 (0.68) | 3 (0.58) | 0.85 |
| Myanmar  (n=386) | 2 (0.52) | 2 (1.22) | 0 (0.00) | 0.10 |
| Philippines  (n=421) | 18 (4.28) | 7 (3.91) | 11 (4.55) | 0.75 |
| Vietnam  (n=3419) | 187 (5.47) | 67 (4.06) | 120 (6.79) | **<0.001** |
|  |  |  |  |  |
| **Playing-physically** |  |  |  |  |
| Bangladesh  (n=1599) | 673 (42.05) | 352 (50.21) | 321 (35.75) | **<0.001** |
| India  (n=5595) | 4768 (85.22) | 2303 (89.79) | 2465 (81.35) | **<0.001** |
| Indonesia  (n=812) | 355 (43.72) | 136 (46.58) | 219 (42.12) | 0.22 |
| Myanmar  (n=386) | 208 (53.89) | 110 (67.07) | 98 (44.14) | **<0.001** |
| Philippines  (n=421) | 205 (48.69) | 105 (58.66) | 100 (41.32) | 0.14 |
| Vietnam  (n=3419) | 1736 (50.78) | 984 (59.60) | 752 (42.53) | **<0.001** |
|  |  |  |  |  |
| **Sleeping in daytime** |  |  |  |  |
| Bangladesh  (n=1599) | 1102 (68.92) | 474 (67.62) | 628 (69.93) | 0.70 |
| India  (n=5595) | 4311 (77.05) | 1921 (74.89) | 2390 (78.88) | **0.034** |
| Indonesia  (n=812) | 214 (26.35) | 81 (27.74) | 133 (25.58) | 0.50 |
| Myanmar  (n=386) | 194 (50.26) | 67 (40.85) | 127 (57.21) | **0.039** |
| Philippines  (n=421) | 297 (70.54) | 120 (67.04) | 177 (73.14) | 0.34 |
| Vietnam  (n=3419) | 805 (23.54) | 358 (21.68) | 447 (25.28) | **0.013** |
|  |  |  |  |  |
| **Watching TV** |  |  |  |  |
| Bangladesh  (n=1599) | 1124 (70.29) | 484 (69.04) | 640 (71.27) | 0.57 |
| India  (n=5595) | 4308 (77.00) | 1981 (77.23) | 2327 (76.80) | 0.59 |
| Indonesia  (n=812) | 298 (36.7) | 106 (36.30) | 192 (36.92) | 0.86 |
| Myanmar  (n=386) | 229 (59.33) | 90 (54.88) | 139 (62.61) | **0.010** |
| Philippines  (n=421) | 322 (76.48) | 144 (80.45) | 188 (77.69) | 0.57 |
| Vietnam  (n=3419) | 1956 (57.21) | 927 (56.15) | 1029 (58.20) | 0.23 |
|  |  |  |  |  |
| **Playing games in TV, Phones, tabs, etc.**  **(Using electronic devices)** |  |  |  |  |
| Bangladesh  (n=1599) | 625 (39.09) | 318 (45.36) | 307 (34.19) | **<0.001** |
| India  (n=5595) | 3718 (66.45) | 1801 (70.21) | 1917 (63.27) | **<0.001** |
| Indonesia  (n=812) | 155 (19.09) | 70 (23.97) | 85 (16.35) | **0.008** |
| Myanmar  (n=386) | 127 (32.90) | 61 (37.20) | 66 (29.73) | **0.034** |
| Philippines  (n=421) | 240 (57.00) | 104 (58.10) | 136 (56.20) | 0.20 |
| Vietnam  (n=3419) | 833 (24.36) | 495 (29.98) | 338 (19.12) | **<0.001** |

*P*-values are generated from Chi test

**Supplemental Table 5. Current concerns by gender among adolescents in Asia Pacific countries during early COVID-19**

|  | Total | Male | Female | *P*-value |
| --- | --- | --- | --- | --- |
|  |  | N=5552 | N=6680 |  |
| **Feeling stressed or isolated** | n (%) | n (%) | n (%) |  |
| Bangladesh  (n=1599) | 1392 (87.05) | 619 (87.30) | 780 (86.86) | 0.79 |
| India  (n=5595) | 2227 (39.80) | 981 (38.25) | 1246 (41.12) | **0.029** |
| Indonesia  (n=812) | 472 (58.13) | 173 (59.25) | 299 (57.50) | 0.63 |
| Myanmar  (n=386) | 111 (28.76) | 52 (31.71) | 59 (26.58) | 0.06 |
| Philippines  (n=421) | 187 (44.42) | 76 (42.46) | 111 (45.87) | 0.68 |
| Vietnam  (n=3419) | 1002 (29.31) | 453 (27.44) | 549 (31.05) | **0.020** |
|  |  |  |  |  |
| **Worry about getting sick** |  |  |  |  |
| Bangladesh  (n=1599) | 626 (39.15) | 270 (38.52) | 356 (39.64) | 0.65 |
| India  (n=5595) | 1505 (26.90) | 656 (25.58) | 849 (28.02) | **0.040** |
| Indonesia  (n=812) | 280 (34.48) | 110 (37.67) | 170 (32.69) | 0.15 |
| Myanmar  (n=386) | 56 (14.51) | 25 (15.24) | 31 (13.96) | 0.72 |
| Philippines  (n=421) | 145 (34.44) | 60 (33.52) | 85 (35.12) | 0.73 |
| Vietnam  (n=3419) | 1467 (42.94) | 691 (41.93) | 776 (43.89) | 0.25 |
|  |  |  |  |  |
| **Concerns for not going to school** |  |  |  |  |
| Bangladesh  (n=1599) | 1244 (77.80) | 530 (75.61) | 714 (79.51) | 0.06 |
| India  (n=5595) | 3843 (68.69) | 1699 (66.24) | 2144 (70.76) | **<0.001** |
| Indonesia  (n=812) | 277 (34.11) | 98 (33.56) | 179 (34.42) | 0.80 |
| Myanmar  (n=386) | 107 (27.72) | 37 (22.56) | 70 (31.53) | 0.05 |
| Philippines  (n=421) | 248 (58.91) | 105 (58.66) | 143 (59.09) | 0.93 |
| Vietnam  (n=3419) | 2048 (59.95) | 961 (58.31) | 1087 (61.48) | 0.06 |
|  |  |  |  |  |
| **Concerns for missing friends** |  |  |  |  |
| Bangladesh  (n=1599) | 1300 (81.30) | 568 (81.03) | 732 (81.51) | 0.80 |
| India  (n=5595) | 3821 (68.29) | 1737 (67.72) | 2084 (68.78) | 0.40 |
| Indonesia  (n=812) | 170 (20.94) | 60 (20.55) | 110 (21.15) | 0.84 |
| Myanmar  (n=386) | 108 (27.98) | 42 (25.61) | 66 (29.73) | 0.37 |
| Philippines  (n=421) | 208 (49.41) | 86 (48.04) | 122 (50.41) | 0.63 |
| Vietnam  (n=3419) | 1521 (44.53) | 755 (45.81) | 766 (43.33) | 0.14 |
|  |  |  |  |  |
| **Concerns for household income / food security** |  |  |  |  |
| Bangladesh  (n=1599) | 957 (59.85) | 414 (59.06) | 543 (60.47) | 0.57 |
| India  (n=5595) | 1601 (28.61) | 697 (27.17) | 904 (29.83) | **0.028** |
| Indonesia  (n=812) | 75 (9.24) | 26 (8.90) | 49 (9.42) | 0.81 |
| Myanmar  (n=386) | 80 (20.73) | 29 (17.68) | 51(22.97) | 0.21 |
| Philippines  (n=421) | 119 (28.27) | 40 (22.35) | 79 (32.64) | **0.020** |
| Vietnam  (n=3419) | 469 (13.73) | 201 (12.20) | 268 (15.16) | **0.012** |
|  |  |  |  |  |
| **Feeling unsafe or insecure** |  |  |  |  |
| Bangladesh  (n=1599) | 475 (29.71) | 202 (28.82) | 273 (30.40) | **0.491** |
| India  (n=5595) | 566 (10.12) | 249 (9.71) | 317 (10.46) | 0.35 |
| Indonesia  (n=812) | 116 (14.29) | 41 (14.04) | 75 (14.42) | 0.88 |
| Myanmar  (n=386) | 26 (6.74) | 16 (9.76) | 10 (4.50) | **0.042** |
| Philippines  (n=421) | 52 (12.35) | 14 (7.82) | 38 (15.70) | **0.015** |
| Vietnam  (n=3419) | 495 (14.49) | 217 (13.17) | 278 (15.72) | **0.034** |

*P*-values are generated from Chi test

**Supplemental Table 6. COVID-19 information sources by gender among adolescents in Asia Pacific countries**

|  | Total | Male | Female | *P*-value |
| --- | --- | --- | --- | --- |
|  | n (%) | n (%) | n (%) |  |
| **Television** |  |  |  |  |
| Bangladesh  (n=1599) | 1408 (88.06) | 610 (87.02) | 798 (88.86) | 0.26 |
| India  (n=5595) | 4401 (78.77) | 2012 (78.44) | 2395 (79.04) | 0.58 |
| Indonesia  (n=812) | 601 (74.01) | 222 (76.03) | 379 (72.88) | 0.33 |
| Myanmar  (n=386) | 290 (75.13) | 120 (73.17) | 170 (76.58) | 0.44 |
| Philippines  (n=421) | 362 (85.99) | 156 (87.15) | 206 (85.12) | 0.55 |
| Vietnam  (n=3419) | 2556 (74.82) | 1209 (73.36) | 1347 (76.19) | 0.06 |
|  |  |  |  |  |
| **Internet/social media** |  |  |  |  |
| Bangladesh  (n=1599) | 751 (46.97) | 396 (56.49) | 355 (39.53) | **<0.001** |
| India  (n=5595) | 2342 (41.86) | 1127 (43.94) | 1215 (40.10) | **0.004** |
| Indonesia  (n=812) | 292 (35.96) | 103 (35.27) | 189 (36.35) | 0.76 |
| Myanmar  (n=386) | 107 (27.72) | 54 (32.93) | 53 (23.87) | **0.050** |
| Philippines  (n=421) | 238 (56.53) | 86 (48.04) | 152 (62.81) | **0.003** |
| Vietnam  (n=3419) | 2307 (67.54) | 1106 (67.11) | 1201 (67.93) | 0.61 |
|  |  |  |  |  |
| **Mobile (phone call/SMS)** |  |  |  |  |
| Bangladesh  (n=1599) | 947 (59.22) | 407 (58.06) | 540 (60.13) | 0.40 |
| India  (n=5595) | 3581 (64.00) | 1677 (65.38) | 1904 (62.84) | **0.048** |
| Indonesia  (n=812) | 132 (16.26) | 51 (17.47) | 81 (15.58) | 0.48 |
| Myanmar  (n=386) | 50 (12.95) | 24 (14.63) | 26 (11.71) | 0.40 |
| Philippines  (n=421) | 70 (16.63) | 25 (13.97) | 45 (18.60) | 0.21 |
| Vietnam  (n=3419) | 1797 (52.61) | 839 (50.91) | 958 (54.19) | 0.06 |
|  |  |  |  |  |
| **Friends** |  |  |  |  |
| Bangladesh  (n=1599) | 665 (41.59) | 300 (42.80) | 365 (40.65) | 0.39 |
| India  (n=5595) | 2991 (53.46) | 1385 (54.00) | 1606 (53.00) | 0.46 |
| Indonesia  (n=812) | 181 (22.29) | 64 (21.92) | 117 (22.50) | 0.85 |
| Myanmar  (n=386) | 98 (25.39) | 39 (23.78) | 59 (26.58) | 0.53 |
| Philippines  (n=421) | 42 (9.98) | 22 (12.29) | 20 (8.26) | 0.17 |
| Vietnam  (n=3419) | 1749 (51.20) | 809 (49.09) | 940 (53.17) | **0.017** |
|  |  |  |  |  |
| **Family** |  |  |  |  |
| Bangladesh  (n=1599) | 945 (59.10) | 377 (53.78) | 568 (63.25) | **<0.001** |
| India  (n=5595) | 3439 (61.47) | 1495 (58.28) | 1944 (64.16) | **<0.001** |
| Indonesia  (n=812) | 305 (37.56) | 106 (36.30) | 199 (38.27) | 0.58 |
| Myanmar  (n=386) | 164 (42.49) | 70 (42.68) | 94 (42.34) | 0.95 |
| Philippines  (n=421) | 115 (27.32) | 56 (31.28) | 59 (24.38) | 0.12 |
| Vietnam  (n=3419) | 1989 (58.23) | 947 (57.46) | 1042 (58.94) | 0.38 |

*P*-values are generated from Chi test
